# Loss of FCN3 links cirrhotic matrix stiffness to YAP activation in HCC

**DOI:** 10.1186/s12967-026-08795-7

**Published:** 2026-08-13

**Authors:** Jia-Mei Luo, Wei Xuan, Guojun Qian, Yiying Tao, Xiaoqiang Wang, Yuqing Chen, Jialin Wu, Zhi-Gang Zhang, Jie Tian

**Affiliations:** 1 Department of Anesthesiology, Renji Hospital, Shanghai Jiao Tong University School of Medicine; Key Laboratory of Anesthesiology (Shanghai Jiao Tong University), Ministry of Education, Shanghai, 200127 China; 2 State Key Laboratory of Systems Medicine for Cancer, Shanghai Cancer Institute, Ren Ji Hospital, School of Medicine, Shanghai Jiao Tong University, 800 Dongchuan Road, Shanghai, 200240 China

**Keywords:** Hepatocellular carcinoma, Cirrhosis, Matrix stiffness, FCN3

## Abstract

**Background:**

Cirrhosis-associated matrix stiffness is a major biophysical driver of hepatocellular carcinoma (HCC). How fibrosis-induced stiffness activates oncogenic YAP signaling remains unclear. We investigated the role of ficolin-3 (FCN3) in stiffness-associated regulation of the O-GlcNAc transferase (OGT)–YAP axis in HCC.

**Methods:**

Public transcriptomic datasets comparing cirrhotic and normal liver tissues (GSE114564), as well as HCC tissues from patients with and without cirrhosis (GSE14520), were analyzed. FCN3 protein expression was evaluated in 67 paired HCC and adjacent non-tumorous tissues, and a subset of 20 matched cases was further analyzed by quantitative reverse-transcription PCR and atomic force microscopy. Controlled-stiffness substrates, FCN3 gain- and loss-of-function models, and murine H22 allograft and human HepG2 xenograft models were used for functional validation. Mechanistic studies included RNA sequencing, cycloheximide chase assays, subcellular fractionation, co-immunoprecipitation, structural prediction, YAP-S127A rescue, Thiamet-G treatment, and YAP knockdown.

**Results:**

FCN3 was downregulated in cirrhotic liver and HCC with cirrhosis. In clinical samples, tumor stiffness inversely correlated with FCN3 expression. FCN3 overexpression curtailed proliferation, clonogenicity, migration, and invasion in vitro and suppressed tumor growth in vivo. Transcriptomics revealed YAP pathway inhibition without changes in upstream kinases. FCN3 did not alter YAP mRNA but reduced YAP protein, increased p-YAP(S127), limited nuclear YAP, and shortened YAP half-life. Co-IP showed FCN3 binds OGT and disrupts OGT–YAP association, decreasing YAP O-GlcNAcylation. YAP knockdown partially attenuated FCN3’s anti-tumor effects.

**Conclusions:**

These findings support a stiffness-associated FCN3–OGT–YAP regulatory model in HCC, in which FCN3 expression is associated with reduced OGT–YAP interaction, decreased YAP O-GlcNAcylation, and restricted YAP nuclear activity. Further studies are required to establish direct molecular competition and its independence from canonical Hippo–LATS signaling.

**Supplementary Information:**

The online version contains supplementary material available at https://doi.org/10.1186/s12967-026-08795-7.

## Introduction

Hepatocellular carcinoma (HCC) accounts for approximately 90% of primary liver cancers and remains the third leading cause of cancer-related mortality worldwide [1–3]. The global burden of HCC is projected to rise continuously, with a possible 1.4 million individuals diagnosed in 2040 [4, 5]. Despite advances in systemic therapies [6, 7], the prognosis of patients with advanced HCC remains poor, underscoring the need for a better understanding of the mechanisms driving hepatocarcinogenesis [2, 3, 8–13]. More than 80% of HCCs arise in cirrhotic livers caused by chronic hepatitis B or C virus infection, alcohol-associated liver disease, or non-alcoholic fatty liver disease (NAFLD) [2, 3, 14, 15]. Beyond serving as a premalignant condition, cirrhosis profoundly remodels the hepatic microenvironment through excessive extracellular matrix (ECM) deposition, chronic inflammation, vascular remodeling, and progressive matrix stiffening [16–18]. Increasing evidence suggests that these biomechanical alterations actively contribute to malignant transformation rather than simply reflecting disease progression [19–21].

Among the pathological features of cirrhosis, increased matrix stiffness has emerged as a key biophysical regulator of HCC initiation and progression [16, 22–24]. Clinically, liver stiffness measured by elastography is closely associated with HCC risk and poor prognosis [25–28], while experimental studies have shown that stiff matrices promote tumor cell proliferation, invasion, metastasis, stemness, and therapeutic resistance [16, 23, 29, 30]. These effects are mediated through mechanotransduction, whereby cells convert extracellular mechanical cues into intracellular signaling events [20, 31]. Yes-associated protein (YAP), the principal effector of the Hippo pathway, is widely recognized as a central mechanosensor that integrates matrix stiffness with transcriptional programs governing cell proliferation and survival [20, 32–38]. However, accumulating evidence indicates that mechanical activation of YAP cannot be fully explained by the canonical Hippo kinase cascade alone [35, 36, 39, 40]. Mechanical forces can also promote YAP nuclear entry by inducing nuclear flattening and increasing the permeability of nuclear pores [41]. The molecular mechanisms linking cirrhosis-associated matrix stiffness to sustained YAP activation therefore remain incompletely understood.

An additional layer of YAP regulation is provided by crosstalk between phosphorylation and O-GlcNAcylation [33, 42]. O-linked β-N-acetylglucosamine (O-GlcNAc) modification, catalyzed by O-GlcNAc transferase (OGT), has been reported to antagonize YAP phosphorylation at Ser127, thereby enhancing YAP stability, nuclear accumulation, and transcriptional activity [42, 43]. OGT-mediated O-GlcNAcylation has been implicated in metabolic reprogramming and tumor progression in multiple malignancies, including HCC [44–47]. Nevertheless, the endogenous molecules that restrain OGT-mediated YAP activation under pathological mechanical stress remain largely unknown. Identifying such regulators may provide mechanistic insight into how cirrhotic matrix stiffness is coupled to oncogenic signaling.

To identify candidate regulators involved in fibrosis-associated hepatocarcinogenesis, we integrated transcriptomic analyses of cirrhotic liver tissues and cirrhosis-associated HCC with clinical validation in human specimens. These analyses consistently identified ficolin-3 (FCN3) as one of the most significantly downregulated genes. FCN3 is a liver-enriched recognition molecule of the lectin complement pathway and has recently been reported to possess tumor-suppressive properties in HCC [48–50], although its role in mechanotransduction has not been investigated. Here, we show that matrix stiffening suppresses FCN3 expression, thereby promoting OGT-dependent YAP O-GlcNAcylation, reducing Ser127 phosphorylation, and enhancing YAP nuclear localization. These findings identify an FCN3–OGT–YAP signaling axis that links cirrhotic matrix stiffness to YAP activation and provide mechanistic insight into stiffness-driven hepatocarcinogenesis.

## Materials and methods

### Patient samples collection

Tumor and adjacent non-tumorous liver tissues were obtained from patients who underwent surgical resection for HCC at Renji Hospital, Shanghai Jiao Tong University School of Medicine, between 2020 and 2023. All patients were diagnosed with HCC based on pathological examination, and none had received preoperative chemotherapy or radiotherapy. For all 67 cases, representative tumor and adjacent non-tumorous tissues were fixed in 4% paraformaldehyde and subsequently processed for paraffin embedding and immunohistochemical analyses [51]. Among these patients, additional matched fresh tumor and adjacent non-tumorous tissues were available from 20 cases. For these 20 cases, tissues were immediately divided into two portions: one portion was kept unfixed in ice-cold sterile PBS and used for AFM measurements, another portion was snap-frozen in liquid nitrogen and stored at − 80 °C for RNA and protein extraction. Written informed consent was obtained from all participants prior to sample collection. The study protocol was approved by the Ethics Committee of Renji Hospital, Shanghai Jiao Tong University School of Medicine (Institutional Approval Code: KY-2020-185), and conducted in accordance with the Declaration of Helsinki.

### Measurement of tissue stiffness

Atomic force microscopy (AFM) measurements were performed at the Instrumental Analysis Center of Shanghai Jiao Tong University by independent technical personnel according to standardized operating procedures. The AFM operator had no access to FCN3 expression data, clinical outcomes, or study hypotheses during data acquisition. Tumor and adjacent tissues were analyzed in parallel. Regions containing gross necrosis, hemorrhage, or large vascular structures were avoided whenever possible to minimize local mechanical heterogeneity. Raw force–indentation data were subsequently returned to the investigators for downstream analyses.

Human liver tissues, including tumor and matched adjacent non-tumorous regions, were kept in ice-cold sterile phosphate-buffered saline (PBS). Tissue stiffness was quantified using an atomic force microscope (AFM, JPK NanoWizard 4, Bruker, Germany) equipped with an MLCT-O10 cantilever (Bruker) fitted with a 12-µm spherical probe (spring constant 0.10 N/m). Force–distance curves were acquired in PBS at room temperature, and Young’s modulus values were calculated using the Hertz contact model implemented in NanoScope Analysis software (version 1.8). For each specimen, 6–10 independent measurement sites were randomly selected and analyzed, and the mean Young’s modulus was used to represent tissue stiffness. Clinical characteristics of the AFM validation cohort are summarized in Supplementary Table 1.

### Cell lines and cell culture

All human HCC cell lines and the mouse HCC cell line H22 were obtained from FuHeng Cell Center (Shanghai, China). All cell lines were cultured in high-glucose Dulbecco’s modified Eagle’s medium (DMEM, Gibco, Grand Island, NY) supplemented with 10% fetal bovine serum (FBS, Gibco) and 0.5% penicillin/streptomycin (Yeasen, Shanghai, China) in a humidified incubator (37 °C, 5% CO_2_).

### Cell culture on substrates of defined stiffness

Hep3B cells were cultured on commercially available CytoSoft^®^ 6-well plates containing functionalized silicone gel substrates with defined elastic moduli of 0.2 kPa (Cat. No. 5165-5EA) and 32 kPa (Cat. No. 5144-5EA). The plates were manufactured by Advanced BioMatrix (USA) and purchased from Amyjet Scientific (Wuhan, China). Before cell seeding, the substrates were coated with type I collagen at a final concentration of 100 µg/mL, using 3 mL coating solution per well, and incubated at 37 °C for 2 h. After removal of the coating solution, the wells were gently rinsed with sterile PBS. Hep3B cells were then cultured in high-glucose DMEM supplemented with 10% fetal bovine serum and 1% penicillin/streptomycin at 37 °C in a humidified atmosphere containing 5% CO₂. When cells reached approximately 80% confluence, total RNA and protein were collected for analysis of FCN3 expression by quantitative reverse-transcription PCR (qRT-PCR) and immunoblotting, respectively. Three independent biological replicates were performed.

### Cell assays

Cell proliferation assay: 5$\:\times\:$10^3^ HCC cells were seeded in a 96-well plate and cultured for 72 h. At 0 h, 24 h, 48 h and 72 h, cells were incubated with 10 µL of CCK-8 reagent (Yeason, Shanghai, China) for 30 min, and optical density (OD) was measured at 450 nm.

Colony formation assay: 5$\:\times\:$10^3^ HCC cells were seeded in 6-well plates and cultured for 7–10 days. Cells were fixed with 4% paraformaldehyde (PFA) for 20 min, stained with 0.1% crystal violet for 20 min, and washed with PBS.

Wound healing assay: 2$\:\times\:$10^5^ HCC cells were seeded in 12-well plates and cultured until confluent. A straight scratch was made using a 200 µL pipette tip, and images were taken at 0 h, 24 h, and 48 h using a light microscope.

Cell invasion assay: Transwell chambers with an 8 μm pore size (Corning, NY, USA) were used for migration assays. The upper chambers were pre-coated with 0.5 mg/mL Matrigel (BD Biosciences, Shanghai, China) for 30 min at 37 °C. Then, 5$\:\times\:$10^4^ cells suspended in 200 µL serum-free DMEM were seeded into the upper chambers, while 600 µL DMEM containing 10% FBS was added into the lower chambers. After 24 h incubation, cells on the upper surface were removed, and the membranes were fixed with 4% PFA for 20 min and stained with 0.1% crystal violet for 20 min.

### Animal models

All animal studies were approved by the Animal Care and Use Committee of Renji Hospital, Shanghai Jiao Tong University School of Medicine (Institutional Approval Code: RJ2022-1022A), and performed in accordance with the National Institutes of Health (NIH) Guide for the Care and Use of Laboratory Animals and the guidelines of the Administrative Committee of Experimental Animal Care and Use of Shanghai.

8-week-old C57BL/6J mice and 6-week-old nude mice were used to establish the HCC models. All mice were acclimatized for one week before experiments and were then randomly assigned to the indicated experimental groups prior to tumor-cell inoculation. C57BL/6J mice and nude mice were subcutaneously injected with 1$\:\times\:$10^6^ mouse HCC cells and 5$\:\times\:$10^6^ human HCC cells, respectively. Mice were monitored every 2 days. At the endpoint, tumors were harvested, and tumor volumes were calculated using the formula: (V) = (tumor width (W)^2^$\:\:\times\:$ tumor length (L))/2. Animals were euthanized when tumors approached the institutional humane endpoint or if ulceration, impaired mobility, or other predefined humane endpoint criteria were observed. Sample sizes were selected based on our previous experience with similar allograft and xenograft experiments and were comparable to those commonly used in the field [52, 53].

### Cell transfection and generation of stable cell lines

The full-length human *FCN3* coding sequence (NM_003665.4) was cloned into a lentiviral overexpression vector to generate the FCN3 overexpression construct (OV-FCN3). Stable FCN3 knockout (FCN3-KO) cells were generated using a CRISPR/Cas9 lentiviral system containing three independent single-guide RNAs (sgRNAs) targeting human *FCN3*, from which the most efficient sgRNA was selected for subsequent experiments. Stable *YAP1* knockdown cells were established using lentiviral short hairpin RNAs (shRNAs) targeting human *YAP1*. Three candidate shRNAs were initially evaluated, and the construct exhibiting the highest knockdown efficiency was selected for further studies. A non-targeting sgRNA or shRNA served as the corresponding negative control. All lentiviral vectors were designed, packaged, and sequence-verified by OBiO Technology (Shanghai, China).

HCC cells were infected with lentiviral particles in the presence of polybrene. Forty-eight hours after infection, stable cell lines were selected with 4 µg/mL puromycin for OV-FCN3 and FCN3-KO cells or 10 µg/mL blasticidin for shYAP cells. Antibiotic selection was maintained for 3 days, until all non-transduced control cells were eliminated. Stable overexpression, knockout, or knockdown efficiency was confirmed by qRT-PCR and Western blotting before subsequent experiments.

For rescue experiments, a constitutively active YAP(S127A) mutant was generated by site-directed mutagenesis, in which serine 127 was substituted with alanine (TCC→GCC). The mutation was verified by Sanger sequencing. Cells were transiently transfected with the YAP(S127A) expression plasmid or the corresponding empty vector using Lipofectamine 3000 (Invitrogen, Carlsbad, CA, USA) according to the manufacturer’s instructions. Functional assays were performed 48 h after transfection unless otherwise specified.

### Western blot analysis

Cells or tumor tissues were lysed in RIPA buffer (Beyotime, Shanghai, China) containing 1% protease inhibitor cocktail (Sigma-Aldrich, Darmstadt, Germany) and 1% phosphatase inhibitor (Beyotime). Lysates were centrifuged at 12,000 $\:\times\:$g for 15 min at 4 °C, and supernatants were collected. Protein concentrations were determined using a BCA Protein Assay Kit (Thermo Scientific, USA). Equal amounts of protein were separated by SDS-PAGE and transferred onto polyvinylidene fluoride (PVDF) membranes (Millipore, Massachusetts, USA) using a semi-dry Trans-Blot system (Bio-Rad Laboratories, California, USA). Membranes were probed with primary antibodies: anti-FCN3 (Proteintech, Cat# 11867-1-AP, 1:1000), anti-GAPDH (CST, Cat# 2118, 1:2000), anti-Vinculin (Abclonal, Cat# A2752, 1:1000), anti-β-tubulin (CST, Cat# 2146, 1:2000), anti-HA (CST, Cat# 3724, 1:3000), anti-OGT (CST, Cat# 24083, 1:1000), anti-YAP (CST, Cat# 12395, 1:1000), anti-pYAP (Ser127) (CST, Cat# 13008, 1:1000), anti-O-GlcNAc (Abcam, Cat# ab2739, 1:1000), anti-histone H4 (CST, Cat# 13914, 1:1000), anti-β-actin (Abclonal, Cat#AC004, 1:3000), followed by HRP-conjugated secondary antibodies.

### Co-immunoprecipitation

Cells were lysed using lysis buffer for WB/IP (Absin, Cat# abs9116) supplemented with protease and phosphatase inhibitor cocktails (Beyotime, Cat# P1045) and PUGNAc (Sigma, Cat# A7229). Equal amounts of protein (600 µg in 300 µL) were incubated with 6 µL normal rabbit IgG (CST, Cat#2729) or anti-HA (CST, Cat#3724), or with normal mouse IgG (Beyotime, Cat#A7028) or anti-YAP (CST, Cat#12395) overnight at 4 °C. Antibody-protein complexes were captured using protein A/G PLUS- Agarose beads (Santa Cruz, Cat# sc-2003) for 4 h at 4 °C, washed, and analyzed by western blotting.

### RNA isolation and qRT-PCR

Total RNA was extracted using EZ-press RNA Purification Kit (EZ Bioscience, Roseville, MN, USA) according to the manufacturer’s protocol. cDNA was synthesized using PrimeScript™ RT Reagent Kit (Takara, Beijing, China). qRT-PCR was performed using SYBR Green PCR Master Mix on a real-time PCR system. Relative gene expression was normalized to *ACTB* and calculated using the 2^−ΔΔCt^ method. Each sample was analyzed in three technical replicates, and at least three independent biological replicates were performed. The primer sequences used in this study are listed in Supplementary Table 2.

### RNA sequencing and bioinformatic analysis

Total RNA was extracted from three independent biological replicates of control and FCN3-overexpressing Hep3B cells using TRIzol reagent (Life Technologies, Cat# 15596-026). RNA integrity and concentration were assessed prior to library preparation. Poly(A)+ mRNA was enriched using oligo(dT)-coupled magnetic beads, fragmented, and reverse transcribed into first-strand cDNA using random hexamer primers. Double-stranded cDNA was synthesized, end-repaired, A-tailed, ligated to sequencing adapters, size-selected using AMPure XP beads, and PCR-amplified to generate sequencing libraries. Library quality was evaluated by qPCR before sequencing.

Paired-end sequencing was performed on an Illumina platform by Biomarker Technologies (Beijing, China). Raw sequencing reads were subjected to quality control by removing adapter sequences, reads containing > 10% ambiguous bases (N), and low-quality reads with more than 50% of bases having a Phred quality score ≤ 10, yielding high-quality clean reads. Across six libraries (three biological replicates per group), a total of 43.92 Gb of clean data were generated, with Q30 values exceeding 93.7% and mapping rates ranging from 93.8% to 94.6%.

Clean reads were aligned to the human reference genome (Ensembl release 95) using HISAT2 (v2.0.4) [54] with default BMKCloud parameters. Transcript assembly and expression quantification were performed using StringTie [55], and gene expression levels were normalized as fragments per kilobase of transcript per million mapped reads (FPKM). Differentially expressed genes (DEGs) between groups were identified using the BMKCloud RNA-seq analysis pipeline. Genes meeting the predefined statistical thresholds were subjected to downstream functional analyses.

Gene Ontology (GO) enrichment and Kyoto Encyclopedia of Genes and Genomes (KEGG) pathway analyses were performed using the BMKCloud bioinformatics platform (Biomarker Technologies, Beijing, China). Significantly enriched terms and pathways were identified according to adjusted P values generated by the platform.

Gene Set Enrichment Analysis (GSEA) was performed using the BMKCloud bioinformatics pipeline based on the ranked list of all expressed genes generated from differential expression analysis [56]. Rather than relying solely on predefined differentially expressed genes, GSEA evaluates coordinated expression changes across the entire ranked gene list to identify significantly enriched biological pathways. Normalized enrichment score (NES), nominal *P* value, and false discovery rate (FDR) were calculated for each gene set using the default implementation of the BMKCloud workflow. Gene sets with nominal *P* < 0.05 and FDR < 0.25 were considered significantly enriched.

### Public transcriptomic data acquisition and differential expression analysis

RNA-seq data from GSE114564 (GPL11154) and Affymetrix microarray data from GSE14520 (GPL3921) were obtained from the National Center for Biotechnology Information Gene Expression Omnibus (GEO) database [57]. GSE114564 contains 15 normal liver samples and 10 cirrhotic liver samples, while GSE14520 includes 203 HCC samples with cirrhosis (HCC-C) and 18 HCC samples without cirrhosis (HCC-NC). Cirrhosis status and etiological information were defined according to the original clinical annotations provided by the GEO datasets. Samples without available cirrhosis-status information were excluded from this subgroup analysis.

The normalized expression matrices were used for subsequent analyses. For GSE14520, probe identifiers were mapped to gene symbols using the GPL3921 platform annotation, and the median expression value was retained when multiple probes mapped to the same gene. Differential gene expression analysis was performed using “limma” package of R [58]. Genes with a *P* value < 0.05 and an absolute fold change > 1.5, corresponding to ∣log_2_FC∣ > 0.585, were retained for candidate screening. Among the downregulated genes, genes were ranked in ascending order according to their log₂FC values, and the top 20 downregulated genes from each dataset were subjected to overlap analysis.

### Single-cell RNA-sequencing analysis

Single-cell RNA-sequencing (scRNA-seq) data were obtained from GSE149614, a published HCC single-cell atlas including primary HCC tumors, portal vein tumor thrombi, metastatic lymph nodes, and matched non-tumorous liver tissues from 10 patients. To focus on FCN3 expression within the primary hepatic microenvironment, only primary HCC and matched non-tumorous liver samples were retained for subsequent analyses. Raw count matrices were analyzed using R packages “Seurat (v.5.1.0)” [59] and “SingleR (v.2.4.1)” [60, 61]. Genes detected in fewer than three cells were excluded. Cells expressing fewer than 200 genes, more than 5000 UMI counts or more than 15% mitochondrial transcripts were removed. The doublet cells were identified and removed using scDblFinder (v1.16.0) [62] with default parameters. After quality-control filtering, a total of 18 samples with 57,223 high-quality cells were retained for downstream analysis. Following quality control, data were normalized using the “NormalizeData” function and scaled using “ScaleData” function in the “Seurat” package. The top 2000 highly variable genes were identified using the “FindVariableFeatures” function. Principal component analysis (PCA) was performed using the top 20 principal components. To minimize inter-sample variability, batch effect correction was performed using “HarmonyIntegration” [63], with individual patient samples treated as independent batches. Cell clustering was conducted using “FindClusters” function (resolution = 0.5) and visualized by Uniform Manifold Approximation and Projection (UMAP).

Cell-type annotation was initially performed using SingleR with the Human Primary Cell Atlas as the reference dataset. Cluster-specific marker genes were identified using the FindAllMarkers function in Seurat based on the Wilcoxon rank-sum test, with thresholds of log2FC > 1, *P* < 0.05 and min.pct > 0.25. The annotation results were subsequently verified and refined according to canonical marker genes curated from CellMarker 2.0 (http://117.50.127.228/CellMarker/) [64]. To distinguish malignant from non-malignant hepatocytes, the hepatocytes subset was subjected to the same quality-control and data-processing procedures described above and assessed by fastCNV (v.1.14) [65] with normal cells as the diploid reference. CNV burden was summarized by cnv_fraction and aggregated at the seurat_clusters level. Clusters were annotated by integrating sample origin, median CNV burden, and the proportion of CNV-high cells in Supplementary Table 3. FCN3 expression was subsequently compared across annotated cell populations and between malignant and non-malignant hepatocyte subsets.

### Statistical analysis

All experiments were performed at least three times unless otherwise specified. Data are presented as means $\:\pm\:\:$standard deviation (SD). Statistical analyses were performed using GraphPad Prism (v8.2.1). Comparisons between two groups were analyzed using a two-tailed Student’s *t*-test, Mann-Whitney u-test or ANOVA. Survival was compared using log rank testing. *P** <*0.05 was considered statistically significant.

## Results

### Cirrhosis is associated with reduced FCN3 expression and poorer survival in HCC

**Fig. 1:**
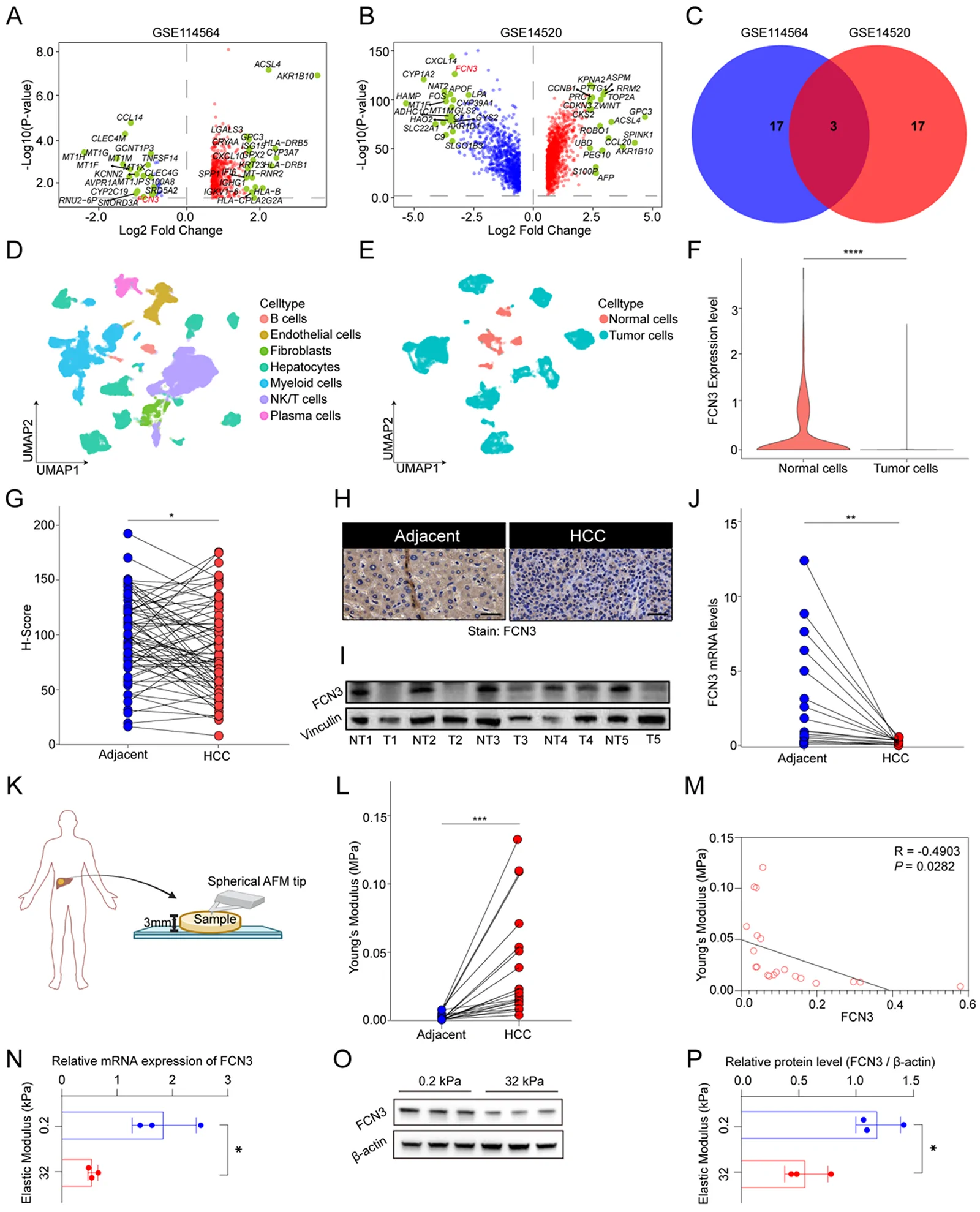
Identification and validation of FCN3 as a cirrhosis-associated gene inversely correlated with liver tissue stiffness. **A**. Volcano plot showing differential gene expression between cirrhotic and normal liver tissues in GSE114564. Red and blue dots represent upregulated and downregulated genes, respectively, selected using a *P* value < 0.05 and an absolute fold change > 1.5, corresponding to ∣log_2_FC∣>0.585. Green dots indicate the top 20 downregulated genes ranked by average log₂ fold change. **B**. Volcano plot showing differential gene expression between hepatocellular carcinoma with cirrhosis (HCC-C) and hepatocellular carcinoma without cirrhosis (HCC-NC) in GSE14520. Red and blue dots represent upregulated and downregulated genes, respectively, identified using a *P* value < 0.05 and an absolute fold change > 1.5, corresponding to ∣log_2_FC∣>0.585. Green dots indicate the top 20 downregulated genes ranked in ascending order according to their log₂ fold-change values. **C**. Venn diagram showing the overlap between the top 20 downregulated genes identified in GSE114564 and GSE14520. Three genes (FCN3, MT1M, and MT1F) were consistently downregulated in both datasets. **D**. UMAP visualization of major cell populations identified from the GSE149614 single-cell RNA-sequencing dataset. **E**. UMAP visualization of hepatocyte subsets classified as normal hepatocytes and malignant hepatocytes based on inferred copy-number variation profiles. **F**. Violin plot showing FCN3 expression in normal and malignant hepatocytes. **G**. Semi-quantitative analysis of FCN3 immunohistochemical staining in paired HCC and adjacent non-tumorous liver tissues (*n* = 67). **H**. Representative immunohistochemical staining of FCN3 in paired HCC and adjacent non-tumorous liver tissues from (**G**). Scale bar = 40 μm. **I**. FCN3 protein expression in 5 pairs of HCC and adjacent non-tumorous liver tissues in Renji’s cohort. **J**. Relative FCN3 mRNA expression in 20 paired HCC and adjacent non-tumorous liver tissues from the Renji Hospital cohort. **K**. Schematic illustration of atomic force microscope (AFM) measurements of tissue stiffness. **L**. Young’s modulus measured in paired HCC and adjacent non-tumorous liver tissues. **M**. Correlation analysis of Young’s modulus and FCN3 expression level in HCC tissues. **N**. Relative FCN3 mRNA expression in HCC cells cultured on collagen-coated substrates with different elastic moduli. **O**. Representative immunoblot showing FCN3 protein expression in HCC cells cultured on soft (0.2 kPa) or stiff (32 kPa) substrates. **P**. Quantification of FCN3 protein expression shown in (**O**). All data are mean ± SD. Graphs were compared using unpaired (**F**, **N** and **P**) or paired (**G**, **J** and **L**) *t*-test. Pearson correlation value was represented by R (M). * *P* < 0.05, ** *P* < 0.01, *** *P* < 0.001, **** *P* < 0.0001

In GSE14520, HCC patients with cirrhosis had significantly poorer survival than those without cirrhosis (Supplementary Figure S1A). To identify cirrhosis-linked transcriptional changes, we compared transcriptomic profiles between cirrhotic vs. normal liver tissues (GSE114564; Fig. 1A) and between HCC with cirrhosis (HCC-C) vs. without cirrhosis (HCC-NC) (GSE14520; Fig. 1B). Three genes, FCN3, MT1M, and MT1F, were among the top downregulated candidates in both datasets. (Fig. 1C and Supplementary Table 4). Kaplan–Meier analysis further showed that higher expression of these genes was associated with a trend toward improved overall survival, with FCN3 showing the strongest association (*P* = 0.06; Supplementary Figures S1B–D), in line with our prior report implicating FCN3 in HCC outcomes [48].

To further define the cellular distribution of FCN3, we analyzed a published single-cell RNA-sequencing dataset. Cell populations were annotated based on canonical lineage markers (Supplementary Figure S1E). FCN3 expression was predominantly detected in non-malignant hepatocytes and was lower in hepatocytes classified as malignant based on inferred CNV profiles. (Figs. 1D–F). Together, these findings identify FCN3 as a hepatocyte-enriched gene that is downregulated in cirrhosis-associated HCC.

### FCN3 reduction is associated with increased tissue stiffness

We next validated FCN3 expression in a clinical cohort comprising 67 paired HCC and adjacent liver tissues. Immunohistochemical staining demonstrated significantly lower FCN3 protein expression in tumor tissues (Fig. 1G and H). Among these patients, additional matched tumor and adjacent non-tumorous tissues were available from 20 cases for molecular and AFM analyses. qRT-PCR and AFM measurements were performed in all 20 pairs, whereas sufficient protein was available from five representative pairs for immunoblotting. Consistent with the immunohistochemical findings, FCN3 mRNA and protein levels were lower in tumor tissues than in matched adjacent tissues (Fig. 1I and J).

Atomic force microscopy (AFM) measurements further showed that tumor tissues exhibited a significantly higher Young’s modulus than the matched adjacent liver tissues (Fig. 1K and L), and FCN3 expression was inversely correlated with tissue stiffness (Fig. 1M). To determine whether matrix stiffness affects FCN3 expression, HCC cells were cultured on collagen-coated substrates with defined elastic moduli relevant to normal and cirrhotic liver stiffness. Cells cultured on the stiffer substrate exhibited significantly lower FCN3 mRNA and protein expression than those cultured on the softer substrate (Figs. 1N–P), suggesting that increased matrix stiffness contributes to FCN3 down regulation.

### FCN3 inhibits in vitro malignant behavior and in vivo growth of HCC

**Fig. 2:**
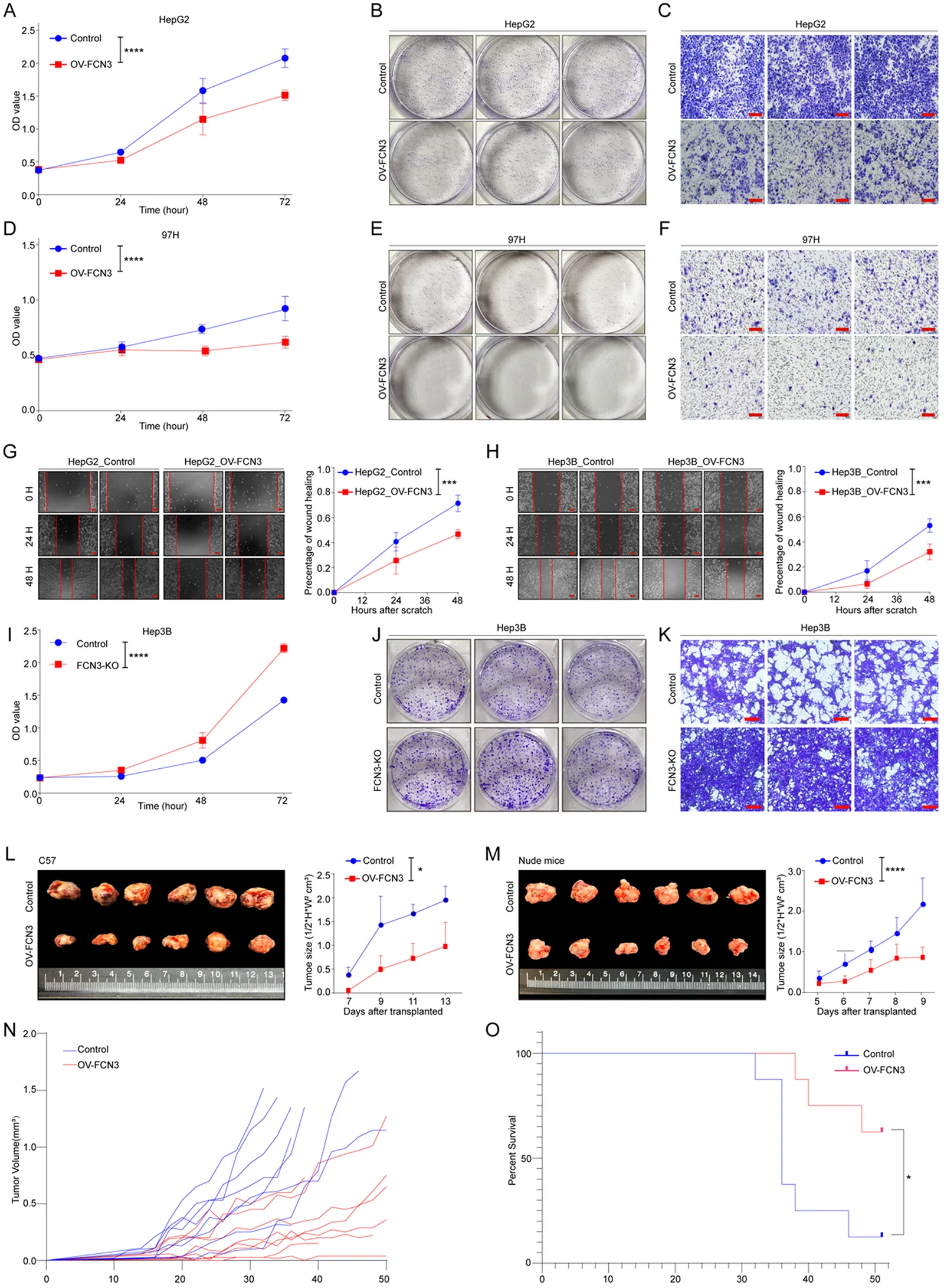
FCN3 inhibits in vitro malignant behavior and in vivo growth of HCC. **A**. CCK-8 assay showing proliferation of HepG2 cells transfected with vector or FCN3 plasmid. **B**. Colony formation assay of HepG2 cells transfected with vector or FCN3 plasmid. **C**. Transwell invasion assay of HepG2 cells transfected with vector or FCN3 plasmid. Scale bars, 200 μm. **D**. CCK-8 assay showing proliferation of MHCC97H cells transfected with vector or FCN3 plasmid. **E**. Colony formation assay of MHCC97H cells transfected with vector or FCN3 plasmid. **F**. Transwell invasion assay of MHCC97H cells transfected with vector or FCN3 plasmid. Scale bars, 200 μm. **G**. Wound-healing assay showing migration of HepG2 cells. Scale bars, 50 μm. **H**. Wound-healing assay showing migration of Hep3B cells. Scale bars, 50 μm. **I**. CCK-8 assay showing proliferation of control and FCN3-knockout Hep3B cells. **J**. Colony formation assay of control and FCN3-knockout Hep3B cells. **K**. Transwell invasion assay of control and FCN3-knockout Hep3B cells. Scale bar = 100 μm. **L**. Representative images and growth curves of subcutaneous tumors derived from control or FCN3-overexpressing H22 cells in C57BL/6 mice (*n* = 10). **M**. Representative images and growth curves of subcutaneous tumors derived from control or FCN3-overexpressing H22 cells in nude mice (*n* = 10). **N**. Individual tumor growth curves of subcutaneous xenografts derived from control or FCN3-overexpressing HepG2 cells in nude mice (*n* = 8). **O**. Kaplan–Meier survival analysis of nude mice bearing control or FCN3-overexpressing HepG2 xenografts. (*n* = 8). All data are mean ± SD. Graphs were compared using two-way ANOVA (**A**, **D**, **G**-**I** and **L**-**M**). Survival was compared using log rank testing (**O**). * *P* < 0.05, *** *P* < 0.001, **** *P* < 0.0001

To investigate the biological function of FCN3 in HCC, we first examined endogenous FCN3 expression in multiple HCC cell lines and immortalized human hepatocyte cell line LO2 (Supplementary Figure S2A). Stable FCN3-overexpressing cell lines were then established using a lentiviral system, and successful overexpression was confirmed by both qRT-PCR and western blotting (Supplementary Figures S2B and S2C). FCN3 gain inhibited the proliferation, colony formation and invasion of HepG2 and MHCC97H, as well as several other cell lines (Figs. 2A-F, Supplementary Figures S2D-G). In addition, FCN3 overexpression suppressed the migratory capability of HepG2 and Hep3B cells (Fig. 2G and H).

To further validate the tumor-suppressive role of FCN3, stable FCN3-knockout (FCN3-KO) cells were generated using a CRISPR/Cas9 strategy. Editing of the FCN3 target locus was verified by Sanger sequencing (Supplementary Figure S2H) and loss of FCN3 protein expression was confirmed by immunoblotting (Supplementary Figure S2I). In contrast to FCN3 overexpression, FCN3 knockout enhanced HCC cell proliferation, colony formation, and invasion (Figs. 2I–K), supporting a growth-suppressive role for endogenous FCN3.

We next examined whether FCN3 affects tumor growth in vivo. Because mice lack a homolog of FCN3, the murine H22 cell line was engineered to stably express human FCN3 (Supplementary Figures S2B and S2C). In immunocompetent C57BL/6 mice, tumors derived from FCN3-overexpressing H22 cells exhibited slower growth than control tumors (Fig. 2L). To determine whether this phenotype depended on host adaptive immunity or on ectopic expression of the human FCN3 transgene in murine cells, we repeated the experiments in immunodeficient nude mice using both H22 and the human HCC cell line HepG2. In both models, FCN3 overexpression consistently suppressed tumor growth (Fig. 2M and N), and also prolonged overall survival (Fig. 2O). These findings indicate that the tumor-suppressive effect of FCN3 is maintained in both murine and human HCC models and is not solely dependent on an intact adaptive immune system.

### FCN3 suppresses YAP transcriptional output by reducing YAP protein abundance

**Fig. 3:**
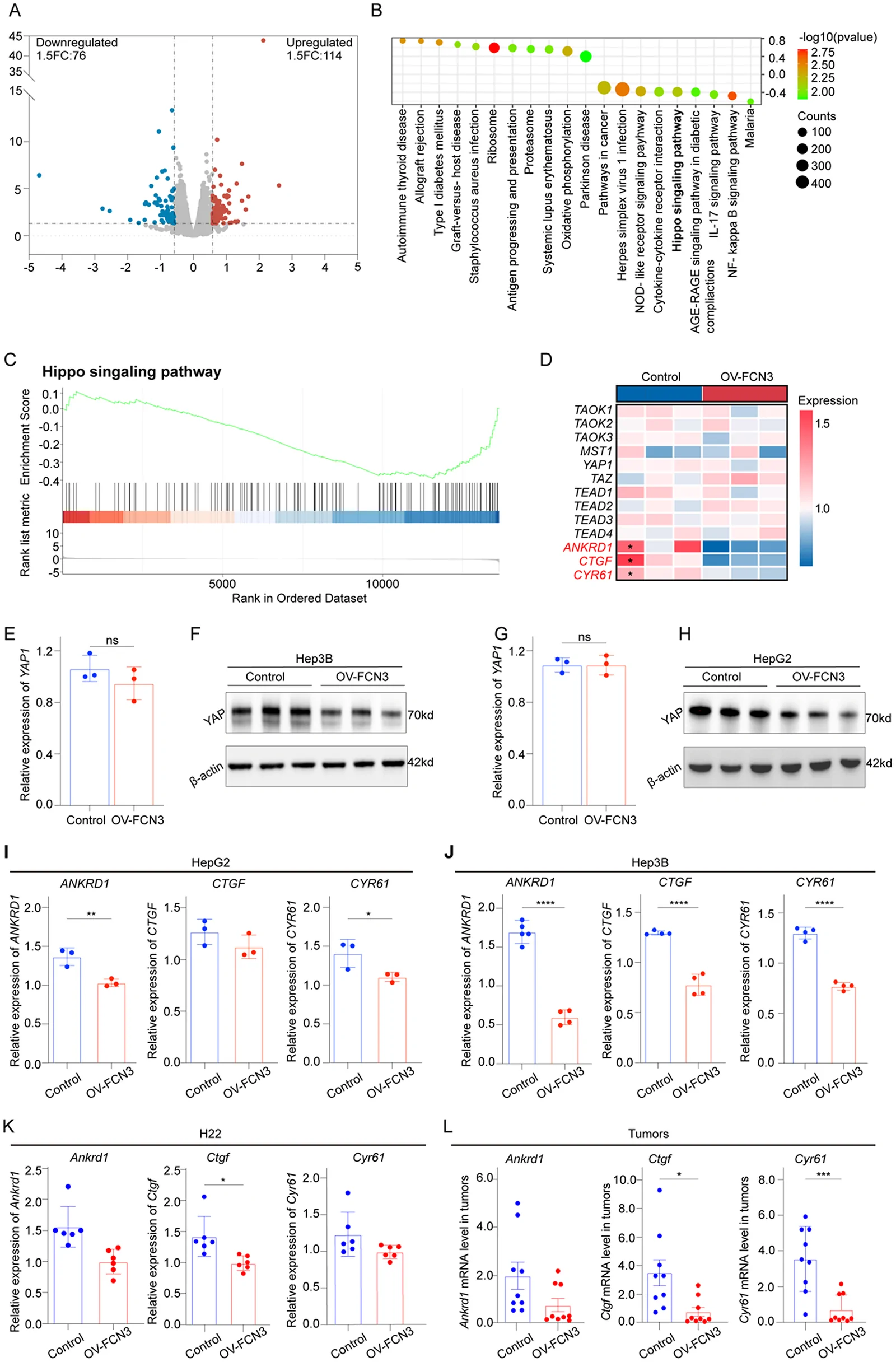
FCN3 overexpression suppresses YAP transcriptional output without altering *YAP1* mRNA expression. **A**. Volcano plot of differentially expressed genes (DEGs) in Hep3B_OV-FCN3 cells compared to Hep3B_Control cells (FCN3 was not shown in the volcano plot due to excessively large fold change). **B**. The top 20 pathways of DEGs in Hep3B_OV-FCN3 cells compared to Hep3B_Control cells based on Kyoto Encyclopedia of Genes and Genomes (KEGG) enrichment analysis. **C**. Gene set differences of Hippo signaling pathway in Hep3B_OV-FCN3 cells compared to Hep3B_Control cells based on gene set enrichment analysis (GSEA). **D**. Heatmap showing the expression of Hippo signaling pathway components and their downstream target genes in control and FCN3-overexpressing Hep3B cells. Each column represents one independent biological replicate. **E**–**F**. Analysis of YAP expression in control and FCN3-overexpressing Hep3B cells. **E**, relative *YAP1* mRNA expression measured by qRT-PCR. **F**, representative immunoblot of YAP protein expression, with β-actin used as the loading control. **G**–**H**. Analysis of YAP expression in control and FCN3-overexpressing HepG2 cells. **G**, relative *YAP1* mRNA expression measured by qRT-PCR. **H**, representative immunoblot of YAP protein expression, with β-actin used as the loading control. **I**–**K**. qRT-PCR analysis of the canonical YAP target genes *ANKRD1*, *CTGF*, and *CYR61* in control and FCN3-overexpressing HepG2 cells (**I**), Hep3B cells (**J**), and H22 cells (**K**). **L**. qRT-PCR analysis of *ANKRD1*, *CTGF*, and *CYR61* expression in subcutaneous H22 allograft tissues derived from control or FCN3-overexpressing cells, corresponding to the tumors shown in Fig. 2L. All data are mean ± SD. Graphs were compared using unpaired *t*-test. * *P* < 0.05, ** *P* < 0.01, *** *P* < 0.001, **** *P* < 0.0001

To investigate the mechanisms underlying FCN3 overexpression induced HCC inhibition effects, total RNAs from FCN3 overexpression Hep3B cells and control cells were subjected to RNA sequencing. In total, 190 genes were found to be differentially expressed (Fig. 3A). KEGG pathway enrichment analysis revealed alterations in multiple signaling pathways (Fig. 3B and Supplementary Table 5). Interestingly, we found that the Hippo signaling pathway, whose major transcriptional effector YAP is known to be regulated by mechanical stress [39, 40, 66], was negatively enriched in OV-FCN3 cells (Fig. 3B and C). This observation prompted us to explore how FCN3 influences Hippo pathway activity. We next analyzed the expression changes of genes involved in the Hippo signaling pathway. RNA-seq analysis identified no significant transcriptional changes in several upstream Hippo pathway components, such as thousand and one amino acid protein kinases (*TAOKs*) and mammalian Ste20-like kinase 1 (*MST1*) (Fig. 3D). However, marked downregulation was observed in its established downstream targets including ankyrin repeat domain protein 1 (*ANKRD1*), connective tissue growth factor (*CTGF*), and cysteine-rich angiogenic inducer 61 (*CYR61*) (Fig. 3D). These findings suggested that FCN3 overexpression was associated with reduced YAP transcriptional output.

We therefore examined the expression level of YAP per se. Interestingly, RNA-seq analysis revealed no significant changes in *YAP1* mRNA levels following FCN3 overexpression (Fig. 3D), which was further confirmed by qRT-PCR in different HCC cell lines (Fig. 3E and G). In contrast, western blot analysis showed a reduction in YAP protein abundance (Fig. 3F and H), indicating that FCN3 regulates the expression of YAP primarily at the post-transcriptional level.

Consistent with reduced YAP activity, FCN3 overexpression decreased the mRNA expression of *ANKRD1*, *CTGF*, and *CYR61* in HepG2, Hep3B, and H22 cells (Figs. 3I–K). Similar reductions were observed in subcutaneous H22 allograft tissues derived from FCN3-overexpressing cells (Fig. 3L). Together, these results indicate that FCN3 suppresses YAP protein abundance and transcriptional activity in HCC cells both in *vitro* and in *vivo*.

### FCN3 reduces YAP stability and nuclear accumulation

**Fig. 4:**
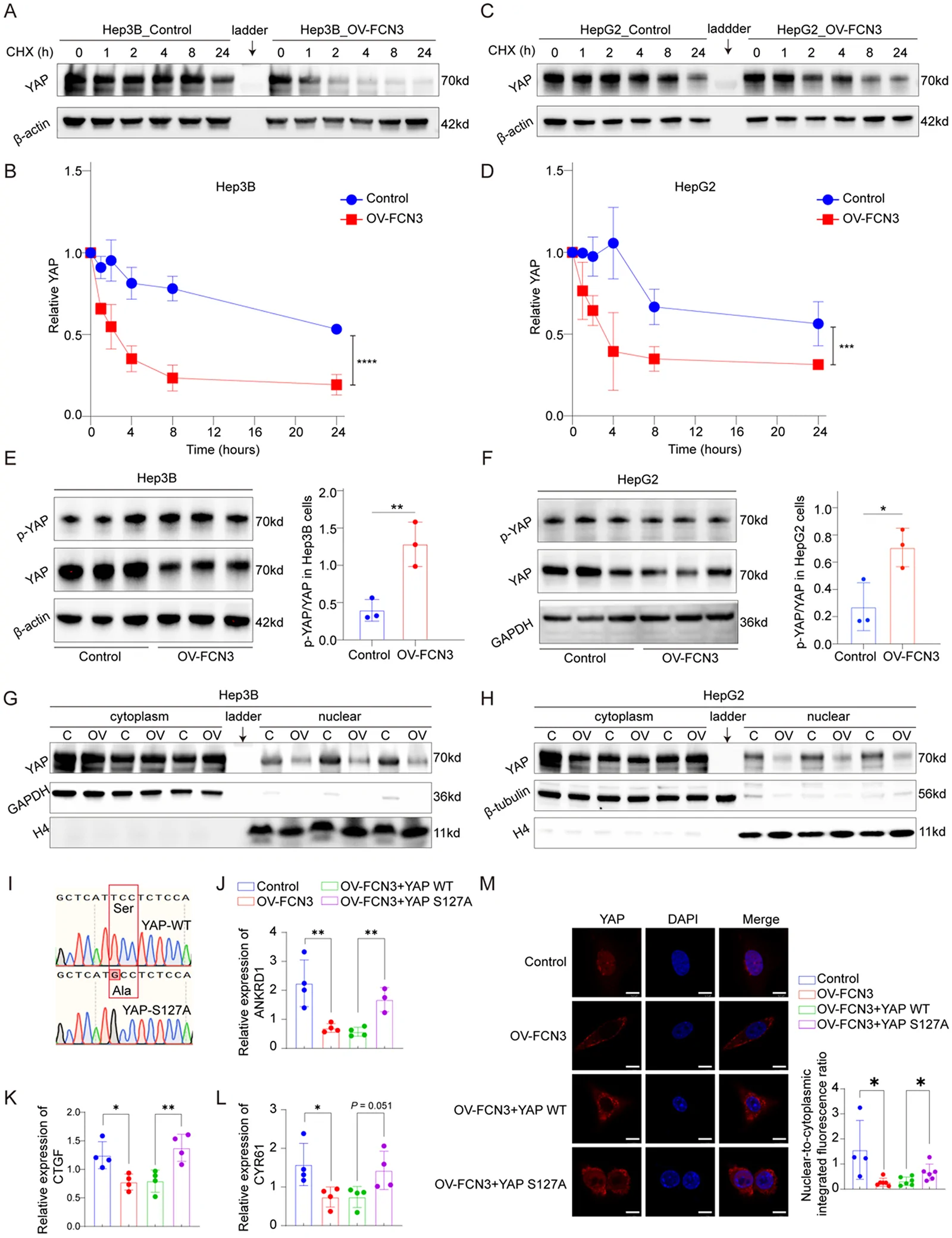
FCN3 promotes YAP degradation and suppresses YAP nuclear localization through a Ser127 phosphorylation–dependent mechanism. **A**-**B**. Cycloheximide (CHX) chase assay showing YAP protein stability in control and FCN3-overexpressing Hep3B cells treated with CHX (50 µg/mL) for 0, 1, 2, 4, 8, or 24 h. Representative immunoblot is shown in (**A**), and quantification of relative YAP protein levels is shown in (**B**). C-D. CHX chase assay showing YAP protein stability in control and FCN3-overexpressing HepG2 cells treated as in (**A**). Representative immunoblot is shown in (**C**), and quantification of relative YAP protein levels is shown in (**D**). **E**. Western blot on lysates of Hep3B_Control and Hep3B_OV-FCN3 cells, blotted with anti-p-YAP(S127), anti-YAP and anti-β-actin. **F**. Western blot on lysates of HepG2_Control and HepG2_OV-FCN3 cells, blotted with anti-p-YAP(S127), anti-YAP and anti-GAPDH. **G**. Western blot on lysates of cytoplasm and nuclear from Hep3B_Control and Hep3B_OV-FCN3 cells, blotted with anti-YAP, anti-GAPDH and anti-H4. **H**. Western blot on lysates of cytoplasm and nuclear from HepG2_Control and HepG2_OV-FCN3 cells, blotted with anti-YAP, anti-β-tubulin and anti-H4. **I**. Sanger sequencing confirming the YAP-S127A mutation. **J**–**L**. RT-qPCR analysis of canonical YAP target genes, including ANKRD1 (J), CTGF (**K**), and CYR61 (**L**), in control cells, FCN3-overexpressing cells, FCN3-overexpressing cells expressing wild-type YAP, and FCN3-overexpressing cells expressing YAP-S127A. **M**. Immunofluorescence analysis of YAP subcellular localization in the indicated groups, with quantification of the nuclear-to-cytoplasmic YAP fluorescence intensity ratio. Scale bar = 10 μm. All data are mean ± SD. Graphs were compared using two-way ANOVA (**B** and **D**) or unpaired *t*-test (**E**-**F** and **J**-**M**). * *P* < 0.05, ** *P* < 0.01, *** *P* < 0.001, **** *P* < 0.0001

To explore how FCN3 regulated the level of YAP expression at the post-transcriptional level in HCC, we first examined YAP protein stability using cycloheximide (CHX) chase assays. We found that FCN3 overexpression significantly reduced the half-life of YAP and promoted its degradation (Figs. 4A-D). Previous studies have shown that YAP S127 phosphorylation promotes its cytoplasmic retention via 14-3-3 binding [67], thereby facilitating S381 phosphorylation-mediated degradation [68]. Therefore, we then analyzed the levels of phosphorylated YAP (p-YAP) at S127. An obvious increase in YAP S127 phosphorylation was observed in FCN3-overexpressing Hep3B cells, but not in HepG2. Notably, in both Hep3B and HepG2 cells, FCN3 significantly increased the p-YAP/YAP ratio, as the total YAP expression level was markedly reduced, suggesting that it promotes a greater proportion of YAP in the phosphorylated state (Fig. 4E and F), which is also consistent with enhanced degradation of YAP. To gain further insight, we next evaluated the subcellular localization of YAP by examining its differential distribution between cytoplasmic and nuclear compartments by western blot. We found that FCN3 markedly decreased YAP levels in the nucleus, while leaving cytoplasmic YAP levels largely unaffected (Fig. 4G and H).

To assess whether Ser127 phosphorylation contributes to FCN3-mediated YAP inhibition, we introduced a phosphorylation-defective YAP-S127A mutant into FCN3-overexpressing HCC cells (Fig. 4I). Compared with wild-type YAP, YAP-S127A partially restored *ANKRD1* and *CTGF* expression and showed a trend toward restoration of *CYR61* expression, under FCN3-overexpressing conditions (Figs. 4J–L). Immunofluorescence analysis further showed that YAP-S127A rescued the FCN3-induced reduction in nuclear YAP localization (Fig. 4M). These data support that Ser127 phosphorylation contributes to FCN3-mediated suppression of YAP nuclear localization and transcriptional output.

### FCN3 disrupts OGT–YAP interaction to suppress YAP O-GlcNAcylation

To elucidate the mechanism underlying the upregulation of YAP phosphorylated at S127 by FCN3, we next analyzed the bulk-seq data following FCN3 overexpression. Gene Ontology molecular function analysis revealed enrichment of carbohydrate-binding-related terms (Supplementary Figure S3A), prompting us to investigate whether glycosylation-associated processes contribute to YAP regulation by FCN3. Given that O-GlcNAcylation has been reported to stabilize YAP and antagonizes its phosphorylation at S127 [42], we examined YAP O-GlcNAcylation following FCN3 overexpression.

**Fig. 5:**
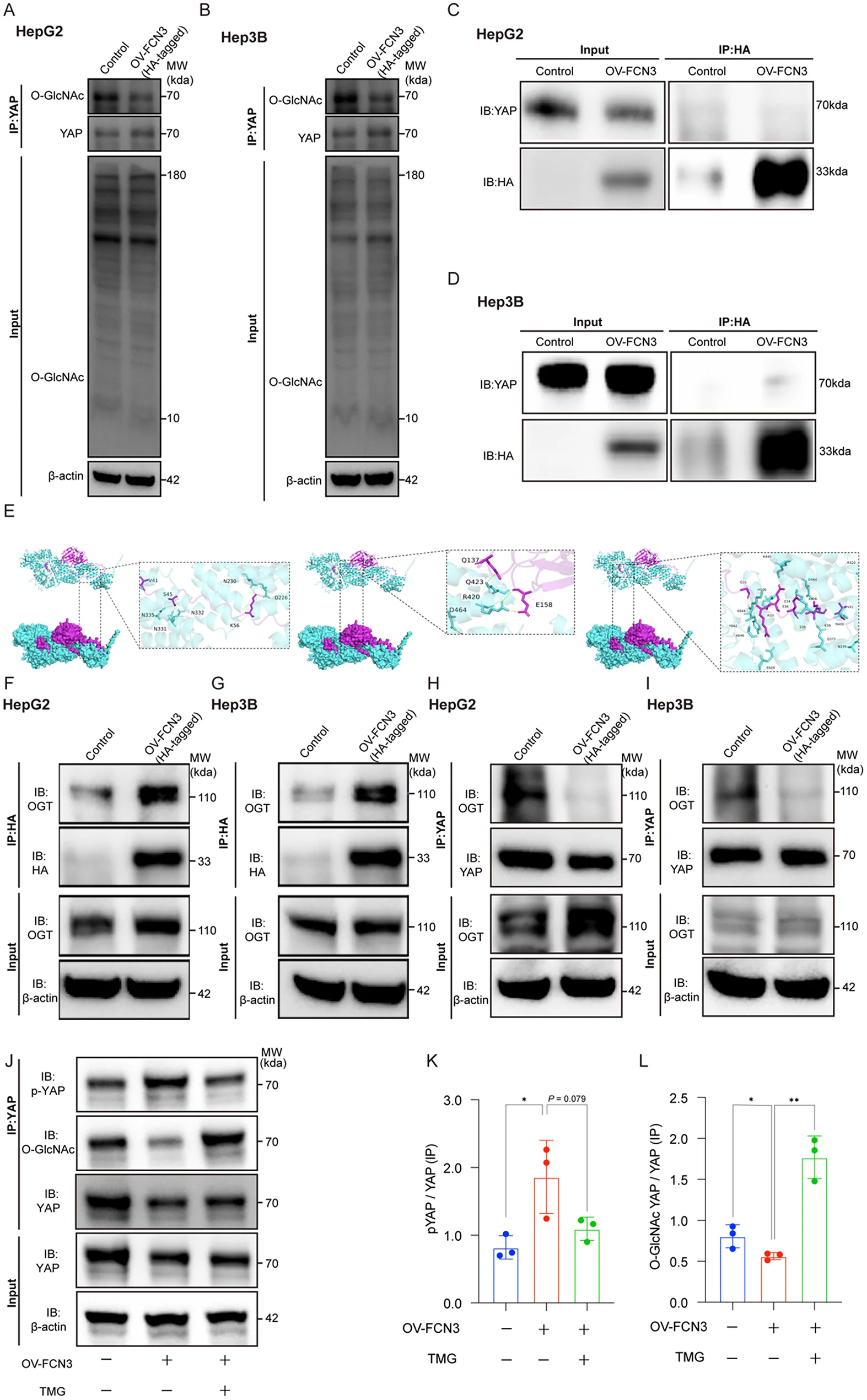
FCN3 disrupts OGT–YAP interaction and suppresses YAP O-GlcNAcylation. **A**-**B**. Immunoprecipitation of endogenous YAP followed by immunoblotting for O-GlcNAc in control and FCN3-overexpressing HepG2 (**A**) and Hep3B (**B**) cells. Total O-GlcNAc and β-actin levels in input lysates are shown below. **C**-**D**. HA was immunoprecipitated in (**C**) HepG2_Control and HepG2_OV-FCN3 cells and (**D**) Hep3B_Control and Hep3B_OV-FCN3 cells, and co-immunoprecipitated YAP was detected by anti-YAP antibody. **E**. AlphaFold3-predicted structures of human OGT (Uniprot ID: O15294-1, color: cyan) and FCN3 (Uniprot ID: O75636-1, color: magenta) interaction complex, OGT and FCN3 are represented as molecular surface. **F**-**G**. HA was immunoprecipitated in (**F**) HepG2_Control and HepG2_OV-FCN3 cells and (**G**) Hep3B_Control and Hep3B_OV-FCN3 cells, and co-immunoprecipitated OGT was detected by anti-OGT antibody. **H**-**I**. YAP was immunoprecipitated in (**H**) HepG2_Control and HepG2_OV-FCN3 cells and (**I**) Hep3B_Control and Hep3B_OV-FCN3 cells, and co-immunoprecipitated OGT was detected by anti-OGT antibody. **J**. Immunoblot analysis of p-YAP(S127), O-GlcNAcylated YAP, and total YAP in control cells, FCN3-overexpressing cells, and FCN3-overexpressing cells treated with Thiamet-G (TMG). **K**–**L**. Quantification of the p-YAP/YAP ratio (**K**) and O-GlcNAc/YAP ratio (**L**) shown in (**J**). All data are mean ± SD. Graphs were compared using unpaired *t*-test (**K** and **L**). * *P* < 0.05, ** *P* < 0.01

Immunoprecipitation of endogenous YAP followed by immunoblotting with an anti-O-GlcNAc antibody showed that FCN3 overexpression markedly reduced YAP O-GlcNAcylation in both HepG2 and Hep3B cells (Fig. 5A and B). In contrast, co-immunoprecipitation (CO-IP) using HA-tagged FCN3 detected no appreciable association between FCN3 and YAP under the conditions tested (Fig. 5C and D), suggesting that FCN3 regulates YAP through another interacting factor.

Because OGT is the sole enzyme responsible for protein O-GlcNAcylation [43], we next investigated whether FCN3 interacts with OGT. AlphaFold3 was used to explore a putative structural basis for FCN3–OGT association [69]. The highest-ranked predicted complex suggested a potential interface between the fibrinogen-like domain of FCN3 and the tetratricopeptide repeat region of OGT (Fig. 5E). This prediction was subsequently evaluated by CO-IP, which supported an association between FCN3 and OGT in HepG2 and Hep3B cells (Fig. 5F and G).

FCN3 overexpression was also associated with reduced co-immunoprecipitation of OGT with YAP (Fig. 5H and I), consistent with reduced YAP O-GlcNAcylation observed following FCN3 overexpression. To determine whether suppression of O-GlcNAcylation contributes to the effects of FCN3 on YAP, FCN3-overexpressing cells were treated with the O-GlcNAcase inhibitor Thiamet-G (TMG), which increases global O-GlcNAcylation. TMG treatment increased YAP O-GlcNAcylation and tended to attenuate the FCN3-induced increase in the p-YAP (S127)/YAP ratio (Figs. 5J-L). Together, these findings support a model in which FCN3 association with OGT may limit OGT–YAP complex formation and thereby reduce YAP O-GlcNAcylation.

### YAP contributes to the FCN3-associated suppression of HCC growth in vitro and in vivo

**Fig. 6:**
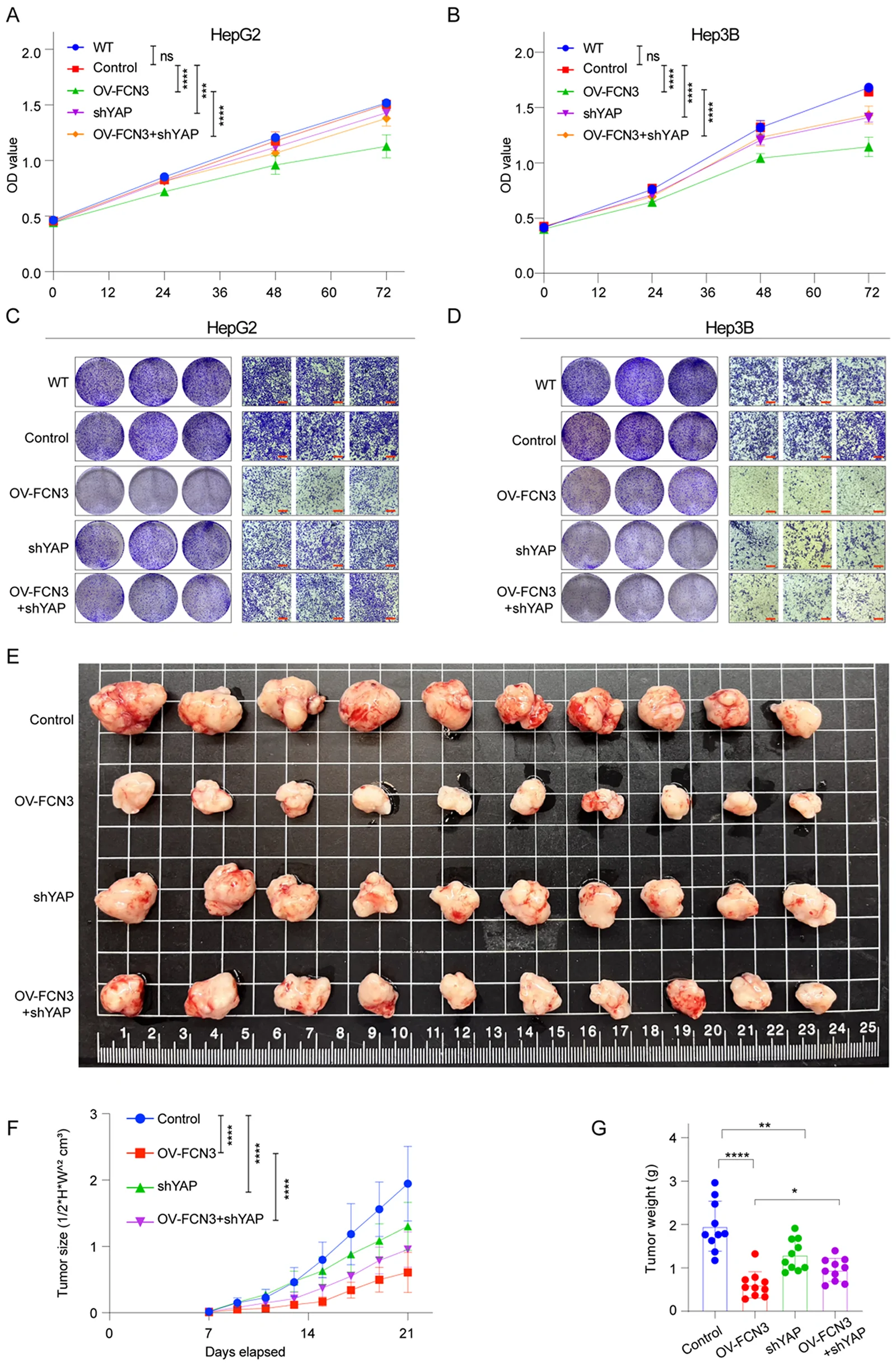
YAP contributes to the FCN3-associated suppression of HCC growth in *vitro* and in *vivo*. **A**-**B**. CCK8 rescue assay in WT group (untreated), Ctrl group (empty vector control), OV-FCN3 group (transduction with FCN3 plasmid), shYAP group (transduction with shYAP lentiviral vectors) and OV-FCN3 + shYAP group (transduction with FCN3 plasmid and shYAP lentiviral vectors). (**A**). HepG2 cells. (**B**) Hep3B cells. **C**-**D**. Colony formation rescue assays (left) and transwell rescue assays (right) in WT group (untreated), Ctrl group (empty vector control), OV-FCN3 group (transduction with FCN3 plasmid), shYAP group (transduction with shYAP lentiviral vectors) and OV-FCN3 + shYAP group (transduction with FCN3 plasmid and shYAP lentiviral vectors). (**C**). HepG2 cells. (**D**) Hep3B cells. Scale bars, 200 μm. **E**. Subcutaneous xenograft tumors from nude mice with HepG2 cells mentioned above. **F**. Tumor growth curves of nude mice subcutaneous xenograft models with HepG2 cells mentioned above. **G**. Tumor weights of nude mice subcutaneous xenograft models with HepG2 cells mentioned above. All data are mean ± SD. Graphs were compared using two-way ANOVA (**A**, **B** and **F**) or one-way ANOVA (**G**). * *P* < 0.05, ** *P* < 0.01, **** *P* < 0.0001

To further investigate whether YAP mediates the tumor-suppressive effects of FCN3, we constructed shYAP lentiviral vectors and validated the knockdown efficiency of YAP using qRT-PCR and western blotting (Supplementary Figures S4A-D). The shYAP-2 virus, which exhibited the highest knockdown efficiency, was selected to generate HCC cell lines with simultaneous FCN3 overexpression and YAP knockdown (HepG2 OV-FCN3 + shYAP and Hep3B OV-FCN3 + shYAP). CCK-8 and colony formation assays revealed that FCN3 overexpression alone inhibited tumor proliferation, while YAP knockdown alone also demonstrated a moderate tumor-suppressive effect. However, when YAP was knocked down, the inhibitory effect of FCN3 overexpression on HCC cell proliferation was partially abrogated (Fig. 6A and B). Similar results were observed in transwell assays (Fig. 6C and D). Moreover, in *vivo* subcutaneous tumor experiments using nude mice demonstrated that YAP knockdown weakened the tumor-suppressive effects of FCN3 (Figs. 6E-G). These findings indicate that YAP expression is at least partially required for the tumor-suppressive effects of FCN3.

## Discussion

Liver cirrhosis is characterized by progressive ECM remodeling and tissue stiffening, creating a biomechanical microenvironment that promotes hepatocarcinogenesis [19, 20, 23]. Although YAP has long been recognized as a central mechanotransducer in liver cancer [32–38, 70, 71], the endogenous mechanisms linking fibrosis-associated matrix stiffness to sustained YAP activation remain incompletely understood. Here, we show a stiffness-associated FCN3–OGT–YAP regulatory model in which FCN3 over-expression is accompanied by reduced YAP O-GlcNAcylation and nuclear signaling.

Mechanical properties of the fibrotic liver are increasingly recognized as active regulators of tumor initiation rather than passive consequences of chronic injury [20, 23, 72]. Growing evidence indicates that increased matrix stiffness actively drives malignant progression through YAP/TAZ-dependent mechanotransduction, metabolic reprogramming, and stromal remodeling [29, 73–77]. In agreement with this concept, FCN3 expression was inversely associated with tissue stiffness in clinical specimens and declined when HCC cells were exposed to substrates mimicking cirrhotic stiffness. These findings suggest that matrix stiffness contributes to HCC progression, at least in part, through suppression of FCN3 expression.

YAP activity is traditionally regulated through the canonical Hippo kinase cascade, in which phosphorylation at Ser127 promotes 14-3-3 binding, cytoplasmic retention and subsequent degradation [32–34, 66–68]. In addition to phosphorylation-dependent control, YAP activity is also regulated by O-GlcNAcylation. OGT-mediated O-GlcNAcylation has been reported to antagonize Ser127 phosphorylation, thereby enhancing YAP stability and nuclear activity [39, 40]. Our findings suggest that FCN3-associated regulation of YAP may involve altered OGT-mediated O-GlcNAcylation. The rescue experiments further support a functional role for OGT-mediated O-GlcNAcylation in FCN3-dependent YAP regulation. Restoration of O-GlcNAcylation by TMG partially relieved the inhibitory effect of FCN3 on YAP, whereas disruption of Ser127 phosphorylation by the YAP-S127A mutant attenuated FCN3-mediated nuclear exclusion of YAP. Together, these observations are consistent with a model in which FCN3 limits YAP activity by modulating the balance between O-GlcNAcylation and Ser127 phosphorylation.

Previous studies have implicated FCN3 as a tumor suppressor in HCC through mechanisms involving activation of the p53 pathway or regulation of ferroptosis [49, 50]. Our findings expand these observations by placing FCN3 within the context of liver mechanobiology. Importantly, YAP knockdown only partially recapitulated the effects of FCN3, suggesting that YAP is an important, but not exclusive, downstream effector of FCN3. It is therefore conceivable that multiple tumor-suppressive pathways converge downstream of FCN3, with the relative contribution of each pathway depending on the biological context. Further studies will be required to determine how FCN3 coordinates these distinct signaling networks during hepatocarcinogenesis.

The present findings also have potential translational implications. FCN3 expression was consistently reduced from cirrhotic liver to cirrhosis-associated HCC and inversely associated with tissue stiffness, suggesting that FCN3 may serve as an indicator of biomechanical remodeling during hepatocarcinogenesis. Moreover, our data identify the FCN3–OGT–YAP axis as a potential therapeutic target for fibrosis-associated HCC. Rather than directly inhibiting YAP, which remains pharmacologically challenging [78], restoration of FCN3 activity or selective disruption of the OGT-YAP interaction may therefore provide an alternative strategy for limiting stiffness-driven YAP activation [79].

Several limitations should be acknowledged. First, although hydrogel culture recapitulates key mechanical features of the cirrhotic microenvironment, it cannot fully reproduce the complexity of fibrosis in vivo. Validation of the FCN3–OGT–YAP axis in fibrosis-driven orthotopic or genetically engineered mouse models will therefore be important in future studies. Second, the subcellular context underlying the observed FCN3–OGT association warrants further investigation. In addition, the molecular mechanisms by which matrix stiffness suppresses FCN3 expression and how the FCN3–OGT–YAP axis integrates with other tumor-suppressive pathways remain to be elucidated. Addressing these questions will further refine our understanding of mechanotransduction during fibrosis-associated hepatocarcinogenesis.

In summary, our study reveals an unexpected role for FCN3 in coupling the biomechanical changes associated with cirrhosis to YAP activation in HCC. Loss of FCN3 promotes OGT-mediated YAP O-GlcNAcylation, reduces Ser127 phosphorylation, and enhances YAP nuclear signaling, thereby facilitating hepatocarcinogenesis. These findings provide mechanistic insight into fibrosis-associated liver cancer and highlight the FCN3–OGT–YAP axis as a mechanistic pathway warranting further preclinical investigation.

## Supplementary Information

Below is the link to the electronic supplementary material.

Supplementary Material 1

Supplementary Material 2

Supplementary Material 3

Supplementary Material 4

Supplementary Material 5

Supplementary Material 6

Supplementary Material 7

## Data Availability

The datasets used and/or analyzed during the current study are available from the corresponding author upon reasonable request.
