# Supplementary material for: Loss of FCN3 links cirrhotic matrix stiffness to YAP activation in HCC
Source: J Transl Med. 2026 Aug 13;24:1386. doi: 10.1186/s12967-026-08795-7 (PMC13737631; doi:10.1186/s12967-026-08795-7)
Supplement: Supplementary file 1 [file 12967_2026_8795_MOESM2_ESM.docx]

Supplementary Table 1. Clinicopathological characteristics of the HCC cohort used for atomic force microscopy analysis

| Characteristics | Value |
| --- | --- |
| Number of patients  Male sex  Age | 20  12 (60%)  56 (36-85) |
| Cirrhosis | 16 (80%) |
| HBV-related | 12 (60%) |
| HCV-related | 1 (5%) |
| Other etiologies | 7 (35%) |
| Tumor size | 4.2 $\pm$1.8 cm |
| Vascular invasion | 5 (25%) |
| Child-Pugh A | 8 (40%) |
| Child-Pugh B | 12 (60%) |
| Child-Pugh C | 0 |
