# Supplementary material for: Loss of FCN3 links cirrhotic matrix stiffness to YAP activation in HCC
Source: J Transl Med. 2026 Aug 13;24:1386. doi: 10.1186/s12967-026-08795-7 (PMC13737631; doi:10.1186/s12967-026-08795-7)
Supplement: Supplementary file 2 [file 12967_2026_8795_MOESM3_ESM.docx]

Supplementary Table 2. Primer sequences used for quantitative reverse-transcription PCR

| Gene | Species | Forword primer (5’-3’) | Reverse primer (5’-3’) |
| --- | --- | --- | --- |
| *FCN3* | Human | TCCGCTCTTGGTCCTCCTA | TGTTGCTTGAATCGTGGTCAG |
| *YAP1* | Human | CTGAACAGTGTGGATGAGATGG | CAGGAATGGCTTCAAGGTAGTC |
| *ANKRD1* | Human | TCATTACGGAACCTGTGGATGT | AGTGGATGGCTGTGGATTCAA |
| *CTGF* | Human | CGATAAGACCTTCCGCCAGA | GCTCCTCCTCATCCTCATTGT |
| *CYR61* | Human | CCACACGAGTTACCAATGACAA | GGAACCGCATCTTCACAGTC |
| *ACTB* | Human | CGTGCGTGACATTAAGGAGAA | GGAAGGAAGGCTGGAAGAGT |
| *Yap1* | Mouse | CGGCAGGCAATACGGAATATC | CGCTGTCTGTGCTCTCATCT |
| *Ankrd1* | Mouse | GGTGAGGACTGGTCATTACGA | GCTATGCGAGAGTTCTTGTAGG |
| *Ctgf* | Mouse | CAGCGGTGAGTCCTTCCAA | ATGTGTCTTCCAGTCGGTAGG |
| *Cyr61* | Mouse | CCACACGAGTTACCAATGACAA | AACCGCATCTTCACAGTTCTG |
| *Actb* | Mouse | AGACCTCTATGCCAACACAGT | TCCTGCTTGCTGATCCACAT |
