# Supplementary material for: Loss of FCN3 links cirrhotic matrix stiffness to YAP activation in HCC
Source: J Transl Med. 2026 Aug 13;24:1386. doi: 10.1186/s12967-026-08795-7 (PMC13737631; doi:10.1186/s12967-026-08795-7)
Supplement: Supplementary file 3 [file 12967_2026_8795_MOESM7_ESM.docx]

**Supplementary Figure 1. Survival analyses and validation of cell-type annotation in the single-cell RNA-sequencing dataset.**

A. Kaplan-Meier analysis of overall survival in HCC patients stratified by cirrhosis status. HCC-NC: HCC patients without cirrhosis, HCC-C: HCC patients with cirrhosis,

B. Kaplan-Meier analysis of overall survival in HCC patients stratified by FCN3 expression status, cutoff = 7.07.

C. Kaplan-Meier analysis of overall survival in HCC patients stratified by MT1F expression status, cutoff = 9.16.

D. Kaplan-Meier analysis of overall survival in HCC patients stratified by MT1M expression status, cutoff = 6.18.

E. Dot plot showing canonical lineage-marker expression across major cell populations in the GSE149614 single-cell RNA-sequencing dataset.

**Survival differences were assessed using the log-rank test.**

**Supplementary Figure 2.** **Validation of FCN3 overexpression and knockout in HCC cells.**

**A. Western blot analysis of endogenous FCN3 expression in HCC cell lines and the immortalized human hepatocyte cell line LO2.**

**B.** **Western blot analysis of FCN3 protein expression in HCC cell lines transfected with vector or FCN3 plasmid**.

**C.** **qRT-PCR analysis of FCN3 mRNA expression in HCC cell lines transfected with vector or FCN3 plasmid.**

D. **EdU assay of Hep3B cells transfected with vector or FCN3 plasmid**. Scale bars, 400 μm.

E. Quantification of EdU-positive cells shown in (D).

**F.** CCK-8 assay showing proliferation of Hep3B cells **transfected with vector or FCN3 plasmid**.

G. **Transwell invasion assay of** Hep3B cells **transfected with vector or FCN3 plasmid**. Scale bars, 200 μm.

H. Sanger sequencing validation of CRISPR/Cas9-mediated FCN3 knockout in Hep3B cells.

**I. Western blot validation of FCN3 knockout in Hep3B cells.**

**All data are mean ± SD. Graphs were compared using unpaired *t*-test (C and E) or two-way ANOVA (F). ** *P* < 0.01, *** *P* < 0.001, **** *P* < 0.0001**

**Supplementary Figure 3**. **The top 20 pathways of DEGs in Hep3B-ovFCN3 cells compared to Hep3B-Ctrl cells based on GO-MF enrichment analysis.**

**Supplementary Figure 4. YAP knockdown is verified in HCC cells at mRNA and protein levels.**

A-B. qRT-PCR analyzed YAP expression of (A) HepG2 cells and (B) Hep3B cells transfected with vector or sh-YAP.

C-D. Western blot analyzed YAP expression of (C) HepG2 cells and (D) Hep3B cells transfected with vector or sh-YAP.

**All data are mean ± SD. Graphs were compared using one-way ANOVA (A and B), **** *P* < 0.0001.**
